# The Role of Systemic Therapies in the Treatment of Grades 1-4 Gliomas

**DOI:** 10.7759/cureus.70532

**Published:** 2024-09-30

**Authors:** Jan Stępka, Mariusz Dotka, Maciej Kosiński, Piotr Suchecki, Maciej Hobot, Igor Piotrowski

**Affiliations:** 1 Oncology, Poznan University of Medical Sciences, Poznań, POL; 2 Medical Physics, Greater Poland Cancer Centre, Poznań, POL

**Keywords:** astrocytoma, central nervous system, chemotherapy, glioblastoma multiforme, glioma, oligodendroglioma, systemic therapy

## Abstract

The primary treatment for gliomas typically involves tumor resection followed by adjuvant radiotherapy, with increasing emphasis on chemotherapy and molecularly targeted drugs. This study aimed to review and summarize the literature on the systemic therapy of malignant gliomas. Chemotherapy may be considered in grades 2 and 3 gliomas, especially when mutations in 1p19q-codeletion are detected. The beneficial impact of adding chemotherapy to radiotherapy (PCV: procarbazine, lomustine, vincristine) has also been demonstrated. In grade 4 glioblastoma multiforme (GBM), wild-type isocitrate dehydrogenase (IDH) status showed the best treatment outcomes with temozolomide (TMZ) in patients with O-6-methylguanine-DNA methyltransferase (MGMT) promoter methylation. Prolonging adjuvant TMZ therapy improves treatment outcomes compared to the standard 6-cycle adjuvant therapy. Bevacizumab (BEV) monotherapy can improve progression-free survival and maintain the initial quality of life. Despite advancements in GBM treatment, outcomes remain unsatisfactory, with a median survival of 14-16 months. Further research is still needed regarding the systemic treatment of central nervous system gliomas.

## Introduction and background

Gliomas are a diverse group of tumors that arise from glial cells in the central nervous system (CNS), representing 26.3% of all primary brain and CNS tumors diagnosed in the United States. Among them, glioblastomas are the most common and aggressive subtype, accounting for 50.9% of all malignant brain tumors [1]. Gliomas can occur at any age, but their incidence peaks in adults between the ages of 45 and 64 years. They are more common in males, with a higher incidence rate compared to females. Additionally, non-Hispanic populations have a higher incidence of gliomas compared to Hispanic populations​ [1].

The prognosis for gliomas varies greatly depending on the tumor's grade, with low-grade gliomas generally having a better outlook. In contrast, glioblastomas are highly aggressive, with only 35.7% of patients surviving five years after diagnosis [1]. The epidemiology of gliomas, particularly glioblastomas, underscores their significant impact on public health due to their high mortality and aggressive progression.

The current classification of gliomas is governed by the fifth edition of the World Health Organization's Central Nervous System Tumor Classification (WHO CNS5). It considers tumor malignancy grade, histopathological type, and subtype, as well as molecular biomarkers [2].

Of paramount clinical significance is the assessment of glioma malignancy, relying on the evaluation of five histopathological criteria, assigning a grade from 1 to 4, where grade 1 is the least aggressive and grade 4 is the most aggressive glioma (Table 1) [2].

**Table 1:** WHO glioma grading and characteristics.

Grades	Histopathological characteristics	Aggressiveness
1	Does not meet grade 2 criteria.	Least aggressive
2	Moderately increased cellularity, sporadic nuclear atypia, absence of mitotic activity or presence of 1 mitosis, no necrosis.	Low-to-moderate aggressiveness
3	Increased cellularity, distinct nuclear atypia, prominent mitotic activity, no necrosis, no endothelial proliferation.	Moderately aggressive
4	High cellularity, marked nuclear atypia, high mitotic activity, presence of necrosis, endothelial proliferation.	Highly aggressive

The WHO does not recommend specific molecular assessment for individual lesions unless necessary for diagnosing a distinct tumor type or subtype [2-4]. The classification, adhering to traditional neuro-oncological divisions, assigns one or more possible WHO grades to glioma types (Table 2) [2].

**Table 2:** Grades of selected glioma types according to WHO CNS.

Glioma type	WHO grade
Polymorphous low-grade neuroepithelial tumor of the young	1
Diffuse astrocytoma, MYB- or MYBL1-altered	1
Myxopapillary ependymoma	2
Supratentorial/posterior fossa ependymoma	2, 3
Oligodendroglioma, IDH-mutant, and 1p/19q-codeleted	2, 3
Pleomorphic xanthoastrocytoma	2, 3
Astrocytoma, IDH-mutant	2, 3, 4
Glioblastoma, IDH-wildtype	4
Diffuse hemispheric glioma, H3 G34-mutant	4

Various therapeutic approaches are currently employed depending on the glioma grade, and the customization of systemic therapy based on specific mutations becomes increasingly important. Treatment outcomes remain unsatisfactory, particularly for grade 4 gliomas, where median survival is estimated at 11-12 months despite optimal treatment [5]. This study aimed to review the literature and summarize knowledge regarding systemic therapy for malignant gliomas. We performed a comprehensive literature review using the PubMed electronic database to identify the most significant studies related to the systemic treatment of gliomas, particularly in grades 2-4. Our search strategy included keywords related to glioma subtypes, molecular markers (such as IDH mutations and 1p/19q co-deletions), and systemic therapies including therapeutics like temozolomide, PCV, and bevacizumab. The search was supplemented by reviewing the bibliographies of key articles to ensure a thorough inclusion of relevant clinical trials and updated treatment protocols.

## Review

Grade 1 gliomas

In the treatment of grade 1 gliomas, systemic therapy is not employed. Instead, surgical treatment and radiotherapy are utilized. Therefore, their treatment will not be considered in this review [6].

Grades 2 and 3 gliomas

Given the similar characteristics of WHO grade 2 and 3 gliomas and their frequent conflation in numerous studies, we have opted to discuss these two types and their respective treatments within a unified section. This category encompasses glioma types such as ependymoma, oligodendroglioma, pleomorphic xanthoastrocytoma, and isocitrate dehydrogenase (IDH)-mutant astrocytoma, as delineated in Table 2. These tumors are characterized by their ability for extensive infiltration and malignancy, which leads to disease progression in subsequent recurrences [6].

Research indicates that molecular markers such as IDH mutations (IDHmt) and 1p19q deletion (1p19q-codeletion) provide more precise prognostic information for patients with gliomas than previous methods, which relied largely on histopathological features. IDH1 and IDH2 are enzymes that play a critical role in cellular metabolism. Mutations in these genes are frequently found in low-grade gliomas, such as astrocytomas and oligodendrogliomas, and they cause the production of an oncometabolite, 2-hydroxyglutarate (2-HG) [7]. This oncometabolite interferes with normal cellular function and promotes tumor growth by altering gene regulation and cell differentiation. Tumors with IDHmt and 1p19q-codeletion are termed "molecular oligodendrogliomas" and tend to have a favorable prognosis. Those with IDHmt but without the 1p19q-codeletion are referred to as “diffuse astrocytomas with IDHmt” or “molecular astrocytomas,” with an intermediate prognosis. In contrast, tumors without IDH mutations (IDHwt) are termed “diffuse astrocytomas with IDHwt” or “molecular glioblastomas,” and they generally behave more aggressively, similar to glioblastomas, which are classified as grade 4 by the WHO [8]. This shift in diagnostic approach has made it more challenging to distinctly differentiate between grade 2 and 3 gliomas, but it has greatly improved prognostic accuracy [6,8].

As per the Polish Society of Clinical Oncology (PTOK) guidelines, the fundamental treatment approach is the surgical removal of the tumor, followed by active observation or radiotherapy in high-risk patients [6]. This approach is also endorsed by the European Association of Neuro-Oncology (EANO) [9]. In cases of recurrence and oligodendroglioma progression, chemotherapy may be considered, particularly in the presence of 1p19q-codeletion mutations [6]. Recent studies suggest that using chemotherapy as an adjunct to first-line treatment alongside radiotherapy offers benefits, including improved progression-free survival, reduced tumor recurrence, and enhanced overall treatment efficacy [10,11]. The preferred chemotherapy options include either temozolomide (TMZ) or a PCV regimen (procarbazine, lomustine, vincristine) [12].

The RTOG9802 study focused on patients with low-grade gliomas, grade II, who were divided into two groups - high and low risk. High-risk patients were randomly assigned to either a group receiving radiotherapy (RT) or a group receiving radiotherapy and chemotherapy with drugs - procarbazine, lomustine, vincristine (RT + PCV). The study results showed that adding chemotherapy significantly extended progression-free survival (PFS) but did not impact overall survival (OS); there were no significant differences in two-year survival. However, three-year and five-year survival rates were 10-15% higher for the RT + PCV group. Shaw et al. suggested that chemotherapy should be considered for low-risk patients who meet certain criteria [10]. A reanalysis of the RTOG9802 study in 2014, after an average observation time of 11.9 years, showed that patients treated with radiotherapy and chemotherapy according to the RT+PCV regimen had a significantly longer OS compared to patients treated with radiotherapy alone (13.3 years vs. 7.8 years), as well as longer PFS (10.4 years vs. 4.4 years) [11]. The RTOG study proved the superiority of combined therapy - RT + PCV over RT monotherapy [10,11].

The bevacizumab (BEV) treatment, often used in recurrent grade 2 and 3 gliomas according to the WHO classification, was evaluated in the TAVAREC study. This study focused on patients experiencing their first recurrence without the coexistence of 1p/19q deletions. The study involved 155 patients, but it was concluded that there is no significant evidence of improved survival time with the combined treatment of BEV and TMZ, compared to therapy with TMZ alone as the OS was reported as 12.9 months in the temozolomide (TMZ) plus bevacizumab (BEV) group and 14.8 months in the TMZ-only group [13].

The German NOA-04 study, which included 318 patients with anaplastic glioma, demonstrated similar effectiveness of chemotherapy and radiotherapy. In this study, chemotherapy regimens TMZ and PCV were used. Patients were divided into the following two groups: (A) receiving radiotherapy and (B) receiving TMZ/PCV chemotherapy. These groups were balanced in terms of the histological characteristics of the tumor. In case of disease progression, patients from group A received one of the chemotherapy regimens, while those from group B received radiotherapy. The median overall survival (OS) was eight years in the radiotherapy group (A) and 6.5 years in the chemotherapy group (B). The median progression-free survival (PFS) was 2.5 years in the radiotherapy group and 2.7 years in the chemotherapy group. OS, PFS, and time to treatment failure were similar in both groups [14].

Molecular analyses conducted within the RTOG 9802, RTOG 9402, and EORTC 26951 studies indicate a varied impact of PCV therapy depending on the genetic profile of tumors. The greatest benefits of therapy are observed in patients with 1p19q deletion, and less so in those without this deletion (1p19q-noncodeletion) [15-17]. In the case of tumors with the wild-type form of IDH, no significant benefits are observed from PCV therapy [15,16]. Similarly, the NOA-04 study showed that among patients with 1p19q-codeletion, monotherapy with PCV is associated with better PFS than monotherapy with TMZ [14]. This suggests that 1p19q-codeletion predicts better outcomes with PCV. However, it is estimated that 30-50% of patients discontinue the full course of PCV due to toxicity. For this reason, some centers prefer to administer TMZ, which is better tolerated [15,17,18]. Additionally, there is a question of whether vincristine significantly improves effectiveness, considering its poor penetration through the blood-brain barrier and high toxicity [19]. Consequently, some centers administer only procarbazine and lomustine, though comparative data on this approach are lacking [20]. The ongoing CODEL study is directly comparing radiotherapy + PCV or TMZ in patients with 1p19q-codeletion (NCT00887146) [21].

The INDIGO trial, a phase 3 study involving 331 patients with residual or recurrent grade 2 IDH-mutant gliomas, demonstrated the efficacy of vorasidenib, an oral, brain-penetrant inhibitor of mutant IDH1 and IDH2. Patients were divided into two groups as follows: those receiving vorasidenib and those receiving a placebo. The groups were balanced in terms of tumor characteristics. The PFS was 27.7 months in the vorasidenib group, compared to 11.1 months in the placebo group. This extension of PFS allows patients to delay treatments such as radiation and chemotherapy, which are associated with cognitive side effects [22]. Vorasidenib's ability to postpone these treatments while maintaining quality of life makes it a critical advancement in the management of low-grade gliomas [23].

There is evidence suggesting that the following treatment methods are suitable for grade 2 or 3 oligodendrogliomas: radiotherapy with PCV [15-17,24], radiotherapy followed by chemotherapy with TMZ, concurrent radiotherapy with TMZ, and radiotherapy with both concurrent and adjuvant use of TMZ [21,25-27].

The primary treatment methods for grade 2 and 3 gliomas focus on surgical resection and radiotherapy, with chemotherapy used upon recurrence [6,9]. Overall, studies indicate a positive impact of systemic treatment in the therapy of these tumors even at early stages [10,11]. The most commonly used chemotherapeutic drugs include TMZ and the PCV sequence (procarbazine, lomustine, vincristine), recommended both as first-line therapy and for recurrent disease [12]. The addition of PCV chemotherapy to radiotherapy has been observed to prolong progression-free survival duration approximately 2.35 times [10,11]. Treatment also utilizes information about the tumor's molecular profile, which has significant prognostic importance and can influence the effectiveness of applied therapies, especially in the context of IDH mutations and 1p/19q deletions [15-17]. Additionally, vorasidenib, an IDH1/IDH2 inhibitor, has shown efficacy in extending progression-free survival in patients with IDH-mutant gliomas, providing an opportunity to delay treatments like radiation and chemotherapy, thus preserving cognitive function [22,23].

Grade 4 gliomas

According to the current WHO CNS classification of gliomas in 2021, among grade 4 diffuse gliomas in adults, we can distinguish grade 4 glioblastoma multiforme (GBM) IDH wild type, diffuse hemispheric glioma with H3 G34 mutation, and in some cases, astrocytoma with IDH mutation, previously diagnosed as a multifocal glioma with IDH mutation. Among these tumors, GBM is by far the most common, being simultaneously the most common primary tumor of the central nervous system [2]. The main predictors of survival for GBM are age, Karnofsky Performance Scale (KPS), and O-6-methylguanine-DNA methyltransferase (MGMT) status [28,29].

First-line treatment for GBM involves macroscopic total resection, if possible, followed by adjuvant radiotherapy with concurrent and adjuvant chemotherapy [30]. Despite advances in concurrent adjuvant therapy, treatment outcomes remain unsatisfactory, with OS of approximately 14 to 16 months. The patient population affected by the disease is highly heterogeneous, and outcomes vary depending on the patient and tumor [31]. The most commonly used systemic therapy for GBM is TMZ, which belongs to the group of alkylating cytostatic drugs that penetrate the blood-brain barrier. It is an oral prodrug that undergoes spontaneous hydrolysis to the active anti-cancer drug in the alkaline environment of gliomas, inhibiting the activity of MGMT, an enzyme involved in DNA repair. This effect can be significant, as there is a link between low MGMT activity in tumor cells and longer survival in GBM patients [32,33]. In the group of patients with MGMT promoter methylation, those treated with temozolomide and radiotherapy had better survival outcomes; their OS was 21.7 months, compared to 15.3 months in patients who only received radiotherapy. In the absence of MGMT promoter methylation, there was a smaller and statistically insignificant difference in survival between the treatment group [34]. The standard chemotherapy regimen consists of six cycles of TMZ. A meta-analysis comparing the results of seven phase 2 clinical trials (including 1018 patients) evaluating the effect of extended adjuvant TMZ therapy (more than six cycles) vs. standard adjuvant TMZ therapy (exactly six cycles) in patients with newly diagnosed GBM showed an increase in PFS (18.8 months vs. 12.1 months, respectively) and OS (24.1 months vs. 18.1 months, respectively) in patients who received extended therapy [35]. This suggests that extending therapy may be clinically beneficial, but additional studies are needed to determine the optimal duration of treatment.

GBM is a highly vascularized tumor and is also rich in vascular endothelial growth factor (VEGF), which promotes the formation of new blood vessels [36]. Therefore, the use of BEV, a humanized monoclonal antibody against VEGF, has been proposed. The results of several randomized controlled trials evaluating the role of BEV in the treatment of newly diagnosed GBM patients did not show a positive impact of BEV on OS but did show an improvement in PFS and maintenance of the initial quality of life. A phase 3 study by Gilbert et al. included patients treated with standard chemoradiotherapy with TMZ, who were assigned to two compared groups as follows: one of them additionally received BEV and the other a placebo. PFS was longer in the BEV group (10.7 months compared to 7.3 months), but no significant difference in OS time was observed between the BEV and placebo groups (median 15.7 and 16.1 months, respectively) [37]. In a similar phase 3 study by Chinot et al., PFS was longer in the BEV group than in the placebo group (10.6 months compared to 6.2 months), but OS did not significantly differ between the groups [38]. In a meta-analysis by Ameratunga et al. (11 studies, 3743 participants), all analyzed studies showed no improvement in overall survival with the addition of anti-angiogenic therapy (eight studies, 2833 participants; high-certainty evidence; three studies, 910 participants; moderate-certainty evidence). However, a pooled analysis from 10 studies (3595 participants) showed improvement in PFS with the addition of BEV [39]. A phase II randomized trial, GENOM 009, which analyzed the effects of adjuvant therapy with the combination of BEV + TMZ, showed longer PFS and OS in the TMZ + BEV arm compared to TMZ monotherapy, although the difference did not reach statistical significance. It was also observed that more patients in the TMZ + BEV group experienced toxicity, but this difference was also not statistically significant [40]. In another clinical trial, higher OS (14.9 vs. 22.1 months) was demonstrated in patients treated with the combination of BEV + TMZ as first-line therapy compared to TMZ monotherapy, especially in patients without MGMT methylation [41].

Recurrent GBM (rGBM) remains a significant difficulty in treatment, with no universally accepted standard of care. Depending on the availability of resources, clinical situation, and local standards, possible therapies include re-treatment with TMZ, monotherapy with nitrosourea alkylating agents (such as lomustine or fotemustine), monotherapy with BEV, or polychemotherapy such as PCV [42].

In clinical trials, BEV has shown satisfactory activity against rGBM, either alone or in combination with irinotecan, with similar PFS rates and median OS was 9.2 months and 8.7 months [43]. A phase 3 trial, EORTC 26101, evaluating the combination of BEV and lomustine in rGBM patients, showed that despite slightly longer PFS, BEV + lomustine treatment did not provide an improvement in survival compared to lomustine monotherapy [44]. However, in the phase II BELOB study, the superiority of lomustine monotherapy over bevacizumab (BEV) monotherapy was demonstrated, as well as the justification for using combined therapy. Patients with the first recurrence of GBM after chemoradiotherapy with TMZ were randomly assigned to one of the following three groups: a group treated with lomustine every six weeks, a group treated with bevacizumab every two weeks, or a group receiving combined treatment with lomustine every six weeks and bevacizumab every two weeks. The median OS was eight months in the lomustine group, eight months in the BEV group, and 12 months in the combined BEV and lomustine group [45].

In several phase 2 trials, combinations of BEV with other drugs such as carboplatin, panobinostat, temsirolimus, sorafenib, and vorinostat were analyzed, but none showed an improvement in outcomes compared to BEV monotherapy [46-50].

Promising results were presented by authors of a retrospective study using a three-drug therapy with TMZ, BEV, and irinotecan (TBI) in the treatment of rGBM patients; the study also included tumor treating fields (TTFields) - electric fields transmitted transdermally to tumors that show antimitotic effects, approved for GBM treatment [51]. In this trial patients were divided into the following two groups based on the chemotherapy received: TBI with TTFields (TBI+T) compared to chemotherapy based on BBC regimens (BEV, BEV + irinotecan, or BEV + lomustine) + TTFields (BBC+T). The median OS and PFS for rGBM patients who received TBI+T were 18.9 and 10.7 months, respectively. In comparison, for patients receiving BBC+T therapy, the median OS was 11.8 months, and PFS was 4.7 months. Although the median PFS results differed significantly by 1.5 months (6.6 vs. 5.1) between the TBI+T and BBC+T groups, the median OS difference of 14.7 months (32.5 vs. 17.8) was more pronounced, with p<0.05. The study was conducted on a small sample of 48 patients, so further prospective studies are needed to confirm the validity of using three-drug therapy in rGBM [51].

Grade 4 gliomas due to their poor prognosis among CNS tumors represent the greatest therapeutic challenge. Therefore, systemic treatment along with radiotherapy as part of concurrent adjuvant therapy is currently an integral part of therapy and the subject of numerous studies. A six-cycle regimen of TMZ is the standard, and extending the duration of therapy seems justified [35]. Additionally, combining BEV with standard TMZ therapy results in prolonged OS and PFS, especially in patients without MGMT methylation [37-41]. Monotherapy with BEV does not currently provide satisfactory results in the case of rGBM, and combining BEV with other drugs has not shown improved survival [44-50]. Further research on combined therapy with TMZ, BEV, and irinotecan (TBI) may offer a chance to increase the effectiveness of rGBM treatment [13-15,22,27-29,31-33,45]. We briefly summarized the available systemic therapies in Table 3.

**Table 3:** Methods of therapy for gliomas with proven clinical efficacy. *Significant benefit when 1p19q-codeletion, minimal when 1p19q-noncodeletion, and none when IDHwt. **Good when IDHmt (IDH1 or IDH2). ***Particularly good when MGMT is present. ****Studies show improvement only in PFS. *****Scientific evidence exists but is not abundant. IDHmt: IDH mutations; IDHwt: IDH without mutation; PFS: progression-free survival

Variables	Grade 1	Grades 2 and 3		Grade 4	
		Oligodendroglioma	Astrocytoma	Glioblastoma multiforme	Recurrent glioblastoma multiforme
Surgical treatment	Resection	Resection	Resection	Resection	Resection
Radiotherapy	To consider	Recommended	Recommended	Recommended	Recommended
Systemic treatment	-	Temozolomide or procarbazine, lomustine, vincristine* or vorasidenib	Temozolomide or procarbazine, lomustine, vincristine* or vorasidenib	Temozolomide** or bevacizumab*** or temozolomide, bevacizumab***	Temozolomide or lomustine/fotemustine or bevacizumab or procarbazine, lomustine, vincristine or temozolomide, bevacizumab, Irinotecan****

Future directions in glioblastoma therapy

Recent advances in the molecular characterization of glioblastoma (GBM) are rapidly being integrated into clinical trials, paving the way for novel therapeutic strategies. Comprehensive profiling of the tumor's genomic, epigenetic, transcriptomic, and proteomic landscapes has provided deeper insights into both glioblastoma's complex biology and the brain microenvironment, allowing for more precise therapeutic targets.

For instance, epidermal growth factor receptor (EGFR) amplification; phosphatase and tensin homolog (PTEN) loss; and MGMT promoter methylation are key molecular alterations currently being explored as therapeutic targets in clinical trials, guiding the development of tailored treatments [52].

Immunotherapeutic approaches, including immune checkpoint inhibitors, chimeric antigen receptor (CAR) T-cell therapy, oncolytic virotherapy, and vaccine-based therapies, are at the forefront of this research [53,54].

Combinatorial therapies are being explored to mitigate side effects and improve the efficacy of these treatments. For example, combining immune checkpoint inhibitors with standard therapies like radiation or chemotherapy has shown promise in preclinical studies [55-59]. Similarly, novel approaches to improve the penetration of therapeutic agents into the tumor are being developed, as the blood-brain barrier (BBB) often limits the effectiveness of systemic treatments. Focused ultrasound therapy, which temporarily disrupts the BBB to allow for better drug delivery, has shown encouraging results in early clinical trials for patients with recurrent GBM [60,61]. This technique could enhance the success of drug therapies in infiltrative regions of the tumor, opening new avenues for treatment combinations that could improve outcomes for GBM patients.

As researchers continue to refine these therapeutic approaches, there is growing optimism that integrating immunotherapy with advanced drug delivery techniques and other novel therapies will yield more effective treatments for GBM.

## Conclusions

The primary method of treating gliomas is resection, with radiotherapy and chemotherapy serving as adjunctive therapies. Systemic treatment is not employed in grade 1 gliomas. For grades 2 and 3, studies have demonstrated the benefit of chemotherapy, particularly significant for patients with the 1p19q-codeletion mutation. The addition of chemotherapy to subsequent radiotherapy has also proven advantageous. In grade 4 glioblastoma multiforme (GBM), the wild-type IDH variant responds relatively well to TMZ, especially in patients with MGMT promoter methylation. For patients without this methylation, combined therapy involving TMZ and BEV appears to be beneficial. There are also benefits observed in extending chemotherapy beyond the standard six cycles. Currently, the treatment of recurrent GBM (rGBM) remains a challenge, with results remaining unsatisfactory. The triple combined therapy involving BEV, irinotecan, and TMZ presents an opportunity to enhance the prognosis in this patient population. The integration of immunotherapy with advanced drug delivery techniques and novel combinatorial therapies holds significant promise for improving treatment outcomes in glioblastoma patients. Recent trials indicate that the choice of systemic treatment should include the genetic makeup of the tumor to ensure the best outcome. Nevertheless, optimization of glioma chemotherapy requires further studies before wider clinical use.

## References

[1] Ostrom QT, Price M, Neff C, Cioffi G, Waite KA, Kruchko C, Barnholtz-Sloan JS (2023). CBTRUS Statistical Report: primary brain and other central nervous system tumors diagnosed in the United States in 2016-2020. Neuro Oncol.

[2] Louis DN, Perry A, Wesseling P (2021). The 2021 WHO Classification of Tumors of the Central Nervous System: a summary. Neuro Oncol.

[3] Marquet G, Dameron O, Saikali S, Mosser J, Burgun A (2007). Grading glioma tumors using OWL-DL and NCI Thesaurus. AMIA Annu Symp Proc.

[4] Sejda A, Grajkowska W, Trubicka J, Szutowicz E, Wojdacz T, Kloc W, Iżycka-Świeszewska E (2022). WHO CNS5 2021 classification of gliomas: a practical review and road signs for diagnosing pathologists and proper patho-clinical and neuro-oncological cooperation. Folia Neuropathol.

[5] Thakkar JP, Dolecek TA, Horbinski C, Ostrom QT, Lightner DD, Barnholtz-Sloan JS, Villano JL (2014). Epidemiologic and molecular prognostic review of glioblastoma. Cancer Epidemiol Biomarkers Prev.

[6] Fijuth J, Dziadziuszko Zespół autorski R, Dziadziuszko R (2013). Nowotwory ośrodkowego układu nerwowego. http://onkologia.zalecenia.med.pl/pdf/zalecenia_PTOK_tom1_02_Nowotwory_OUN_20140807.pdf.

[7] Dang L, White DW, Gross S (2009). Cancer-associated IDH1 mutations produce 2-hydroxyglutarate. Nature.

[8] Brat DJ, Verhaak RG, Aldape KD (2015). Comprehensive, integrative genomic analysis of diffuse lower-grade gliomas. N Engl J Med.

[9] Weller M, van den Bent M, Preusser M (2021). EANO guidelines on the diagnosis and treatment of diffuse gliomas of adulthood. Nat Rev Clin Oncol.

[10] Shaw EG, Wang M, Coons SW (2012). Randomized trial of radiation therapy plus procarbazine, lomustine, and vincristine chemotherapy for supratentorial adult low-grade glioma: initial results of RTOG 9802. J Clin Oncol.

[11] Buckner JC, Pugh SL, Shaw EG (2014). Phase III study of radiation therapy (RT) with or without procarbazine, CCNU, and vincristine (PCV) in low-grade glioma: RTOG 9802 with Alliance, ECOG, and SWOG. J Clin Oncol.

[12] Mair MJ, Geurts M, van den Bent MJ, Berghoff AS (2021). A basic review on systemic treatment options in WHO grade II-III gliomas. Cancer Treat Rev.

[13] van den Bent MJ, Klein M, Smits M (2018). Bevacizumab and temozolomide in patients with first recurrence of WHO grade II and III glioma, without 1p/19q co-deletion (TAVAREC): a randomised controlled phase 2 EORTC trial. Lancet Oncol.

[14] Wick W, Roth P, Hartmann C (2016). Long-term analysis of the NOA-04 randomized phase III trial of sequential radiochemotherapy of anaplastic glioma with PCV or temozolomide. Neuro Oncol.

[15] Bell EH, Zhang P, Shaw EG (2020). Comprehensive genomic analysis in NRG oncology/RTOG 9802: a phase III trial of radiation versus radiation plus procarbazine, lomustine (CCNU), and vincristine in high-risk low-grade glioma. J Clin Oncol.

[16] Cairncross JG, Wang M, Jenkins RB (2014). Benefit from procarbazine, lomustine, and vincristine in oligodendroglial tumors is associated with mutation of IDH. J Clin Oncol.

[17] van den Bent MJ, Brandes AA, Taphoorn MJ (2013). Adjuvant procarbazine, lomustine, and vincristine chemotherapy in newly diagnosed anaplastic oligodendroglioma: long-term follow-up of EORTC brain tumor group study 26951. J Clin Oncol.

[18] Cairncross G, Wang M, Shaw E (2013). Phase III trial of chemoradiotherapy for anaplastic oligodendroglioma: long-term results of RTOG 9402. J Clin Oncol.

[19] Boyle FM, Eller SL, Grossman SA (2004). Penetration of intra-arterially administered vincristine in experimental brain tumor. Neuro Oncol.

[20] Vesper J, Graf E, Wille C, Tilgner J, Trippel M, Nikkhah G, Ostertag C (2009). Retrospective analysis of treatment outcome in 315 patients with oligodendroglial brain tumors. BMC Neurol.

[21] Jaeckle KA, Ballman KV, van den Bent M (2021). CODEL: phase III study of RT, RT + TMZ, or TMZ for newly diagnosed 1p/19q codeleted oligodendroglioma. Analysis from the initial study design. Neuro Oncol.

[22] Mellinghoff IK, Van Den Bent MJ, Blumenthal DT (2023). INDIGO: a global, randomized, double-blinded, phase 3 study of vorasidenib versus placebo in patients with residual or recurrent grade 2 glioma with an IDH1/2 mutation. J Clin Oncol.

[23] O'Leary K (2023). A new targeted therapy for low-grade glioma. Nat Med.

[24] Buckner JC, Shaw EG, Pugh SL (2016). Radiation plus procarbazine, CCNU, and vincristine in low-grade glioma. N Engl J Med.

[25] van den Bent MJ, Baumert B, Erridge SC (2017). Interim results from the CATNON trial (EORTC study 26053-22054) of treatment with concurrent and adjuvant temozolomide for 1p/19q non-co-deleted anaplastic glioma: a phase 3, randomised, open-label intergroup study. Lancet.

[26] Fisher BJ, Pugh SL, Macdonald DR (2020). Phase 2 study of a temozolomide-based chemoradiation therapy regimen for high-risk, low-grade gliomas: long-term results of radiation therapy oncology group 0424. Int J Radiat Oncol Biol Phys.

[27] Hwang K, Kim TM, Park CK (2020). Concurrent and adjuvant temozolomide for newly diagnosed grade III gliomas without 1p/19q co-deletion: a randomized, open-label, phase 2 study (KNOG-1101 study). Cancer Res Treat.

[28] Annavarapu S, Gogate A, Pham T, Davies K, Singh P, Robert N (2021). Treatment patterns and outcomes for patients with newly diagnosed glioblastoma multiforme: a retrospective cohort study. CNS Oncol.

[29] Chambless LB, Kistka HM, Parker SL, Hassam-Malani L, McGirt MJ, Thompson RC (2015). The relative value of postoperative versus preoperative Karnofsky Performance Scale scores as a predictor of survival after surgical resection of glioblastoma multiforme. J Neurooncol.

[30] McMahon DJ, Gleeson JP, O'Reilly S, Bambury RM (2022). Management of newly diagnosed glioblastoma multiforme: current state of the art and emerging therapeutic approaches. Med Oncol.

[31] Stupp R, Hegi ME, Mason WP (2009). Effects of radiotherapy with concomitant and adjuvant temozolomide versus radiotherapy alone on survival in glioblastoma in a randomised phase III study: 5-year analysis of the EORTC-NCIC trial. Lancet Oncol.

[32] Hegi ME, Diserens AC, Godard S (2004). Clinical trial substantiates the predictive value of O-6-methylguanine-DNA methyltransferase promoter methylation in glioblastoma patients treated with temozolomide. Clin Cancer Res.

[33] Esteller M, Garcia-Foncillas J, Andion E (2000). Inactivation of the DNA-repair gene MGMT and the clinical response of gliomas to alkylating agents. N Engl J Med.

[34] Hegi ME, Diserens AC, Gorlia T (2005). MGMT gene silencing and benefit from temozolomide in glioblastoma. N Engl J Med.

[35] Alimohammadi E, Bagheri SR, Taheri S, Dayani M, Abdi A (2020). The impact of extended adjuvant temozolomide in newly diagnosed glioblastoma multiforme: a meta-analysis and systematic review. Oncol Rev.

[36] Yao XH, Ping YF, Chen JH (2008). Glioblastoma stem cells produce vascular endothelial growth factor by activation of a G-protein coupled formylpeptide receptor FPR. J Pathol.

[37] Gilbert MR, Dignam JJ, Armstrong TS (2014). A randomized trial of bevacizumab for newly diagnosed glioblastoma. N Engl J Med.

[38] Chinot OL, Wick W, Mason W (2014). Bevacizumab plus radiotherapy-temozolomide for newly diagnosed glioblastoma. N Engl J Med.

[39] Ameratunga M, Pavlakis N, Wheeler H, Grant R, Simes J, Khasraw M (2018). Anti-angiogenic therapy for high-grade glioma. Cochrane Database Syst Rev.

[40] Balana C, De Las Penas R, Sepúlveda JM (2016). Bevacizumab and temozolomide versus temozolomide alone as neoadjuvant treatment in unresected glioblastoma: the GENOM 009 randomized phase II trial. J Neurooncol.

[41] Hata N, Mizoguchi M, Kuga D (2020). First-line bevacizumab contributes to survival improvement in glioblastoma patients complementary to temozolomide. J Neurooncol.

[42] Kawecki M (2017). The ambiguous role of bevacizumab in progressive glioblastoma - results from
the EORTC 26101 phase III trial. Oncol Clin Pract.

[43] Friedman HS, Prados MD, Wen PY (2009). Bevacizumab alone and in combination with irinotecan in recurrent glioblastoma. J Clin Oncol.

[44] van den Bent M, Gorlia T, Bendszus M (2016). EH1.3 EORTC 26101 phase III trial exploring the combination of bevacizumab and lomustine versus lomustine in patients with first progression of a glioblastoma. Neuro Oncol.

[45] Taal W, Oosterkamp HM, Walenkamp AM (2014). Single-agent bevacizumab or lomustine versus a combination of bevacizumab plus lomustine in patients with recurrent glioblastoma (BELOB trial): a randomised controlled phase 2 trial. Lancet Oncol.

[46] Field KM, Simes J, Nowak AK (2015). Randomized phase 2 study of carboplatin and bevacizumab in recurrent glioblastoma. Neuro Oncol.

[47] Lee EQ, Reardon DA, Schiff D (2015). Phase II study of panobinostat in combination with bevacizumab for recurrent glioblastoma and anaplastic glioma. Neuro Oncol.

[48] Lassen U, Sorensen M, Gaziel TB, Hasselbalch B, Poulsen HS (2013). Phase II study of bevacizumab and temsirolimus combination therapy for recurrent glioblastoma multiforme. Anticancer Res.

[49] Galanis E, Anderson SK, Lafky JM (2013). Phase II study of bevacizumab in combination with sorafenib in recurrent glioblastoma (N0776): a north central cancer treatment group trial. Clin Cancer Res.

[50] Ghiaseddin A, Reardon DA, Massey W (2015). Phase II study of bevacizumab and vorinostat for recurrent glioblastoma. J Clin Oncol.

[51] Lu G, Rao M, Zhu P (2019). Triple-drug therapy with bevacizumab, irinotecan, and temozolomide plus tumor treating fields for recurrent glioblastoma: a retrospective study. Front Neurol.

[52] Brennan CW, Verhaak RG, McKenna A (2013). The somatic genomic landscape of glioblastoma. Cell.

[53] Hamad A, Yusubalieva GM, Baklaushev VP, Chumakov PM, Lipatova AV (2023). Recent developments in glioblastoma therapy: oncolytic viruses and emerging future strategies. Viruses.

[54] Rong L, Li N, Zhang Z (2022). Emerging therapies for glioblastoma: current state and future directions. J Exp Clin Cancer Res.

[55] Liao KL, Huang S, Wu YP (2018). The prognosis for patients with newly diagnosed glioblastoma receiving bevacizumab combination therapy: a meta-analysis. Onco Targets Ther.

[56] Reardon DA, Desjardins A, Vredenburgh JJ (2020). Rindopepimut with bevacizumab for patients with relapsed EGFRvIII-expressing glioblastoma (ReACT): results of a double-blind randomized phase II trial. Clin Cancer Res.

[57] Mathios D, Kim JE, Mangraviti A (2016). Anti-PD-1 antitumor immunity is enhanced by local and abrogated by systemic chemotherapy in GBM. Sci Transl Med.

[58] Hardcastle J, Mills L, Malo CS (2017). Immunovirotherapy with measles virus strains in combination with anti-PD-1 antibody blockade enhances antitumor activity in glioblastoma treatment. Neuro Oncol.

[59] Twumasi-Boateng K, Pettigrew JL, Kwok YY, Bell JC, Nelson BH (2018). Oncolytic viruses as engineering platforms for combination immunotherapy. Nat Rev Cancer.

[60] Idbaih A, Canney M, Belin L (2019). Safety and feasibility of repeated and transient blood-brain barrier disruption by pulsed ultrasound in patients with recurrent glioblastoma. Clin Cancer Res.

[61] Timbie KF, Mead BP, Price RJ (2015). Drug and gene delivery across the blood-brain barrier with focused ultrasound. J Control Release.

